# Complexity Reduction of Rate-Equations Models for Two-Choice Decision-Making

**DOI:** 10.1371/journal.pone.0080820

**Published:** 2013-12-05

**Authors:** José Antonio Carrillo, Stéphane Cordier, Gustavo Deco, Simona Mancini

**Affiliations:** 1 Department of Mathematics, Imperial College London, London, United Kingdom; 2 Univ. Orléans, CNRS, MAPMO, UMR 7349 & FR 2964, Orléans, France; 3 ICREA (Institució Catalana de Recerca i Estudis Avançats), Barcelona, Spain; 4 Computational Neuroscience Unit, Universitat Pompeu Fabra, Barcelona, Spain; National Research & Technology Council, Argentina

## Abstract

We are concerned with the complexity reduction of a stochastic system of differential equations governing the dynamics of a neuronal circuit describing a decision-making task. This reduction is based on the slow-fast behavior of the problem and holds on the whole phase space and not only locally around the spontaneous state. Macroscopic quantities, such as performance and reaction times, computed applying this reduction are in agreement with previous works in which the complexity reduction is locally performed at the spontaneous point by means of a Taylor expansion.

## Introduction

Goal-oriented behavior involves the constant making of decisions between alternative choices. Hence, a thorough understanding of the mechanisms underlying decision-making is fundamental in behavioral neurosciences. During the last years tremendous advances in neurophysiological and theoretical neurosciences have started to reveal the neural mechanisms underlying decision-making. Most of these studies are focused on binary decision-making. In binary decision tasks, the subject is asked to make a choice between two alternatives according to an experimentally defined criterion based on sensory input information. The difficulty of the making of the decision can be manipulated by the level of uncertainty in the discrimination of the sensory input information which can be influenced by regulating the signal to noise ratio. For the same pattern of stimulation the decision of the subject from trial-to-trial is stochastic but such that its trial-to-trial average is determined by the input.

In a wide range of such decision-making experiments, the behavioral response, i.e. performance and reaction times, can be properly described by a simple stochastic diffusion model [1]. In the diffusion model evidences about one or the other choice are accumulated continuously over time until a decision boundary is reached. Hence, it is plausible to think that the underlying neuronal system performs decision-making by accumulation of evidences. Indeed, recent electrophysiological recordings from awake behaving monkeys performing decision-making have shown that trial-averaged spiking activity of some neurons shows a gradual, nearly linear increase shortly after stimulus onset until just before the response is made [2]–[6]. Let us consider for example the so-called random-dot task. The random-dot task is a two-choice visual decision-making task. In this task, the subject is confronted with a visual stimulus consisting of a field of randomly moving dots. On any one trial, a fixed fraction of the dots, determined by the experimentalist, moves coherently in one of two directions. The subject must discriminate in which direction the majority of dots are moving. Electrophysiological recordings from awake behaving monkeys performing this task have shown that trial-averaged spiking activity of neurons in the lateral intraparietal cortex (LIP) reflects the accumulation of information mentioned above [4]–[6]. In fact, LIP cells receive excitatory drive from direction-selective cells of extrastriate visual area MT. The difference in the firing rates of MT cells with opposing preferred directions is a likely measure of the available visual evidence for the direction of coherent motion of the moving dot stimulus, further indicating that the LIP area is involved in this decision-making task.

A computational model of the cortical circuit putatively underlying LIP cell activity has been proposed and studied numerically [7]–[9]. This model was able to explain qualitatively the experimentally observed trial-averaged spiking activity. The model consists of two populations of spiking neurons, within which interactions are mediated by excitatory synapses and between which interactions occur principally through an intermediary population of inhibitory interneurons. Sensory input may bias the competition in favor of one of the populations, potentially resulting in a gradually developing decision in which neurons in the chosen population exhibit increased activity while activity in the other population is inhibited. When the activity of one of the two populations exceeds a pre-defined threshold then a behavioral response is generated. There are two possible mechanisms by which the decision can be made which correspond to two different dynamical regimes. In one mechanism, the appearance of the stimulus destabilizes the so-called spontaneous state, in which all neurons show low activity. In the other mechanism, even after stimulus presentation, the network can still sustain low activity, but random fluctuations eventually drive the collective activity of the network to an activated state, in which a fraction of the neurons fires at a high rate. Let us remark that the behavioral performance of the model (i.e. the fraction of trials in which the ‘correct’ direction is chosen) and corresponding reaction time to make a decision match well the behavioral data from both monkeys and humans performing the moving-dot task [10]. Therefore, we conjecture, that it may be possible to establish a direct link between the neuronal and behavioral correlates of decision-making. Here, we aim to establish this link between the underlying physiology and the observed behavioral response in decision-making tasks by performing a 1D reduction of the dynamics of the neuronal circuit in such a way that we derive from the underlying detailed neuronal dynamics a valid “nonlinear” diffusion model.

In order to perform the explicit link between a neuronal circuit processing decision-making and a diffusion model able to describe the behavioral level, we consider a reduced model of the competitive cortical circuit solving the decision-making problem. In particular, we consider a mean-field reduction consisting of two competing rate models. This system of Langevin equations can be associated with a Fokker-Planck equation for the probability distribution of the activities of the different neuronal populations. The nonlinear nature of the original equations, however, hinders analytical progress in the Fokker-Planck framework. For this reason, the main analysis of such noise driven probabilistic decision-making systems remains based on numerical investigations, which are time consuming because of the need for sufficiently many trials to generate statistically meaningful data.

**Figure 1:**
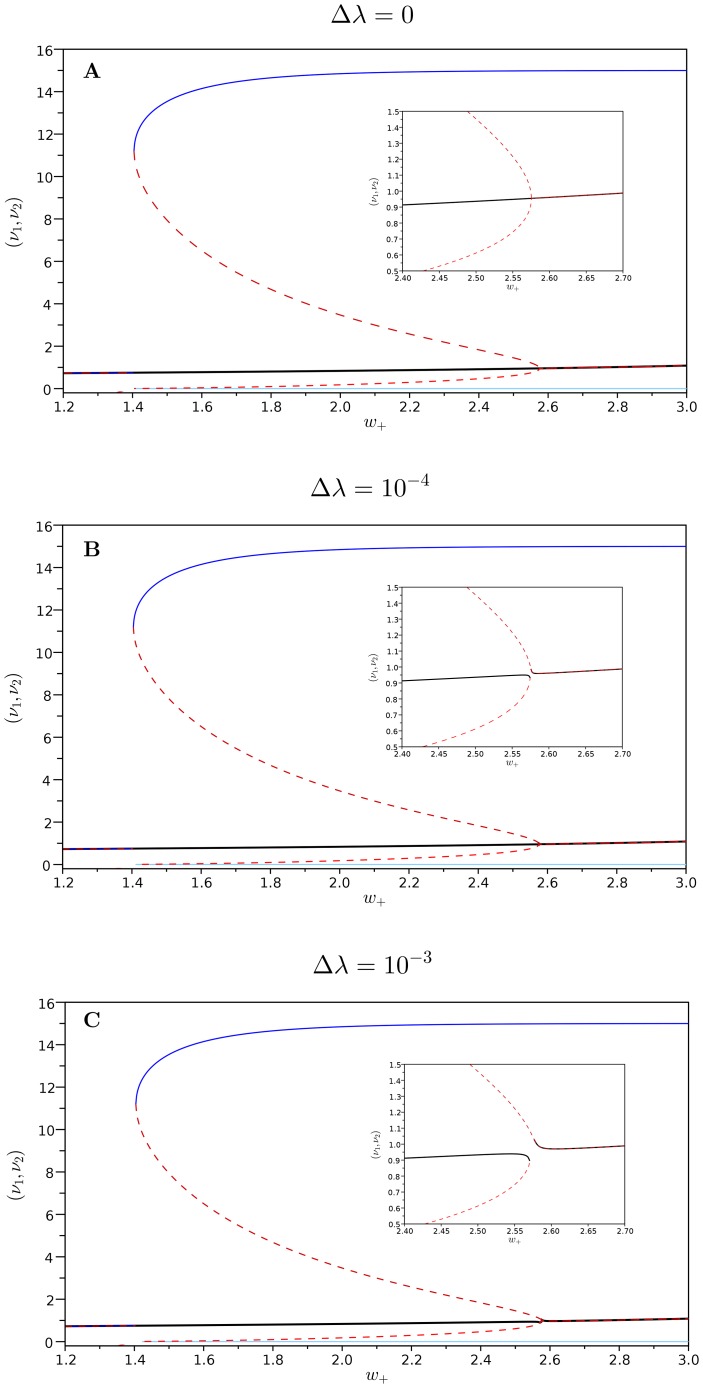
Bifurcation diagrams with respect to 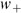. **A**: the bias is 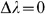; **B**: the bias is 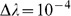; **C**: the bias is 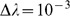. The components of the spontaneous state are traced by the black line. There are other two stable equilibria points which coordinates are represented by the blue and light-blue lines. The red continuous and dashed lines correspond to the unstable equilibrium points. The small picture is a zoom on the bifurcation point.

**Figure 2:**
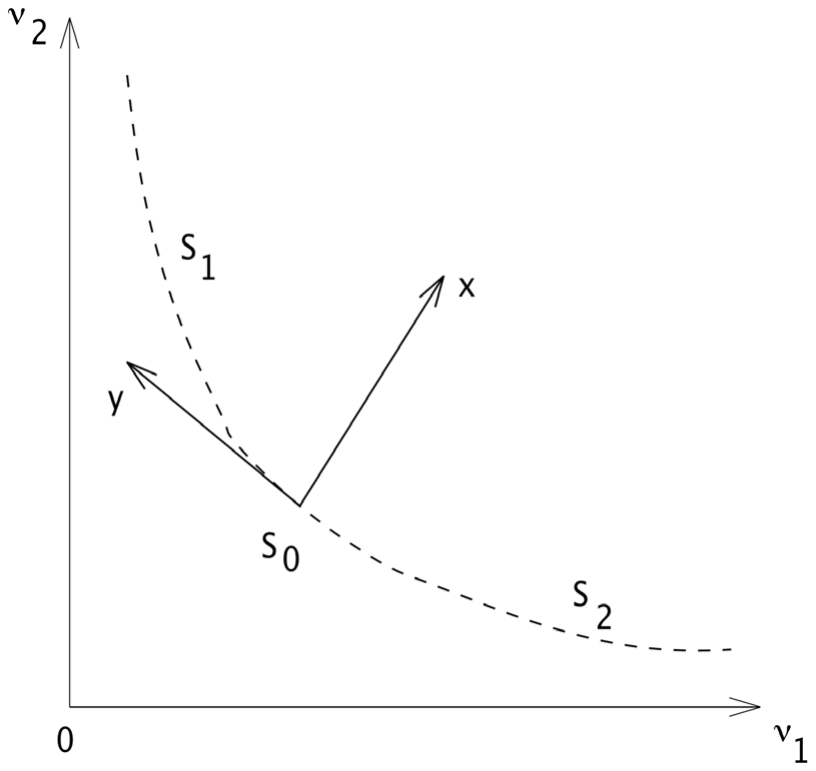
Change of variables. Position of the new coordinate system 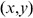 with respect to the initial one 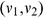. The origin is taken at the central equilibrium point 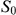 which may stable or unstable accordingly to the value of 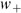. 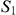 and 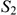 are two others equilibrium points. The dashed line represents the slow manifold on which the reduced dynamics takes place, and it contains all the equilibrium points of the system.

One dimensional Fokker-Planck effective reductions near bifurcation points can be obtained applying the center manifold reduction method as explained in [11]. In this method the projection on the slow manifold is done asymptotically, i.e. order by order in 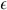, so that the resulting dynamics along the slow manifold does not contain effects of different order. This method is equivalent to the method used in [10], where the authors locally approximate the evolution on the slow manifold at the bifurcation point by Taylor expanding the nonlinearities with respect to the slow variable based on the different time scales. In this way, they obtain an effective potential of degree 4 for the one dimensional Fokker-Planck dynamics on the slow manifold. We propose to build on this strategy by not performing any Taylor expansion and rather approximate the slow manifold in terms of the nonlinearities defining the evolution using the slow-fast character of the dynamical system, see [12]. Our method is similar to the one presented in [11], but once the slow manifold is approximated we then globally project the Fokker-Planck dynamics on it to obtain a one dimensional Fokker-Planck reduction possibly valid beyond the local character of the expansion in [10]. More importantly, we recover an approximated full reduced potential and an explicit formula for the stationary state distribution on the slow manifold. This effective reduced potential covers the other two stable equilibrium points, and not only the central equilibrium point changing its stability character as in [10]. For instance, in the case of the subcritical Hopf bifurcation in Figure 1, we get a three well approximated potential, while the approach in [10] leads to a potential of degree 4 approximating an interval around the central equilibrium point, see ([10], Figure 2). Our strategy is valid as long as the approximated slow manifold lies fully in the relevant biological range of positive rates, this is the reason we restrict the range of the bifurcation parameter 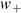 as explained later on.

## Results

We showcase our strategy in a particular example of a neuronal population model with two pools with self-excitation and cross-inhibition as in [13]. The firing rates 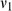 and 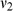 of the neuronal networks are determined by the stochastic dynamical system:
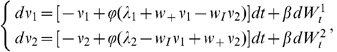
(1)with 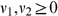. Here the applied stimuli are, 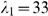, 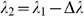, with the bias 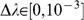, the inhibitory connectivity coefficient is 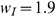, the standard deviation is 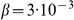, and the excitatory connectivity coefficient 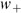 is the bifurcation parameter, its range of values will be discussed later on. Moreover, the sigmoid (response) function 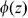 is given by:



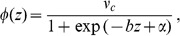
with 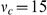, 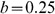, and 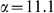.

In terms of 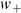 the underlying dynamical system without noise presents a subcritical pitchfork bifurcation whose bifurcation diagram is shown in Figure 1. For values of the excitatory coefficient around 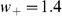 the system pass from a single asymptotically stable equilibria to a situation in which there are three stable (continuous lines) and two unstable equilibria (hashed lines). The second local bifurcation, where the central asymptotic equilibria disappears, happens at around 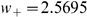 for 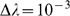.

We next need to find an approximation of the slow manifold joining the spontaneous 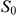 and the decision states 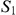 and 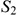, as sketched in Figure 2. This curve is found by introducing the linearization of the dynamical system around the spontaneous equilibria 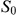 as a new set of variables defined by 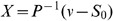, with 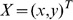, or equivalently 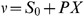 with 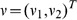. Here, 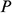 is the matrix diagonalizing the jacobian of the dynamical system (1) at the spontaneous state 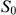, see (2) in the Materials and Methods section for more details, or [14].

**Figure 3:**
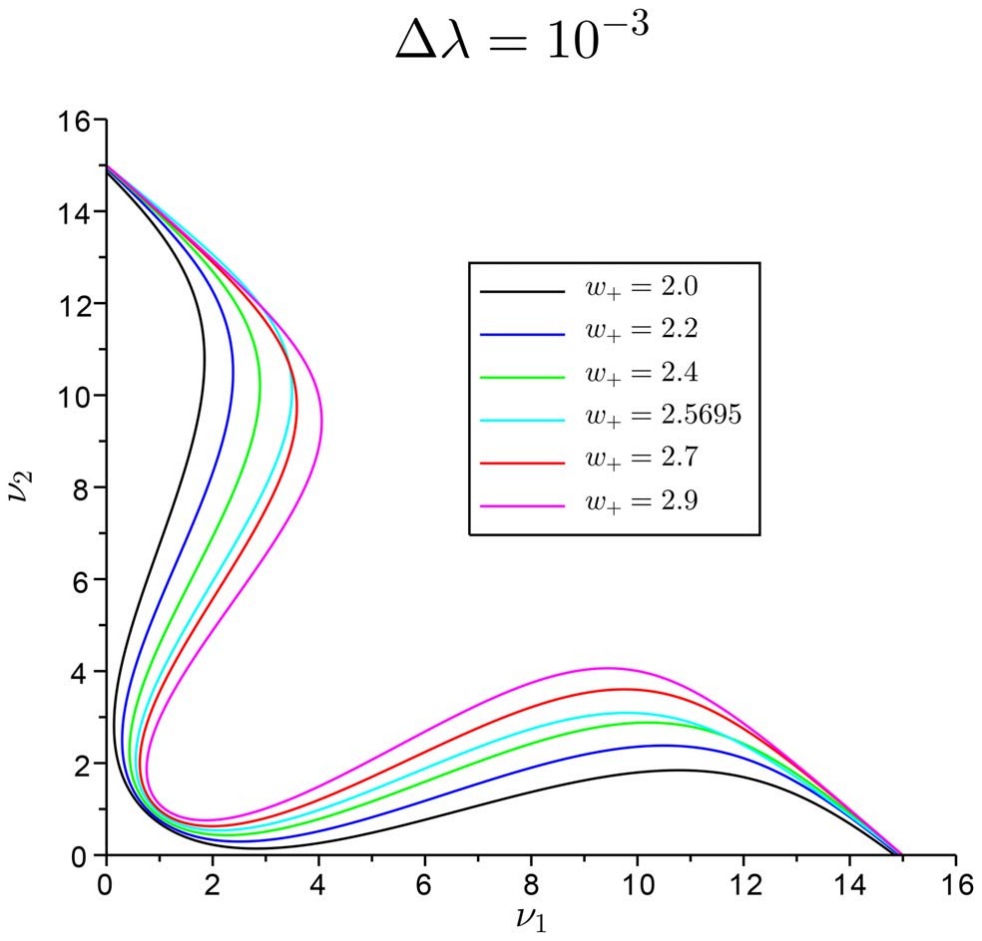
Slow manifold. Variation of the approximated slow manifold represented on the 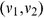 plane for 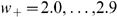 and for the biased case 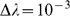. For 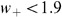 the slow manifold crosses the coordinate axes for small values of 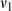 and 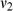, becoming negative. The reduction theory is valid only for ranges of 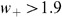.

This change of variables, sketched in Figure 2, is a natural way to introduce slow 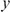 and fast 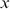 variables in the system determined by the eigenvalues of the linearization at the spontaneous state 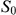. By rewriting the dynamical system above (1) in these new variables together with the new time variable 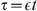, we have an evolution governed by
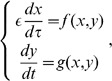
where 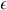 is a small parameter. Assuming that the scaling ratio is zero, 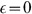, we find the implicit relation 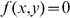 that determines the approximated slow manifold 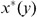. In Figure 3, we plot the curves 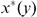 for different values of 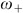 with 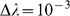. We remark that this restricts the validity of this particular example since these curves exit the set of positive values for the rate variables. This comes from the fact that there are some trajectories of the dynamical system without noise leading to negative values for the rate variables. In this example the approximated curves lie on the positive quadrant for 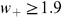 approximately.

**Figure 4:**
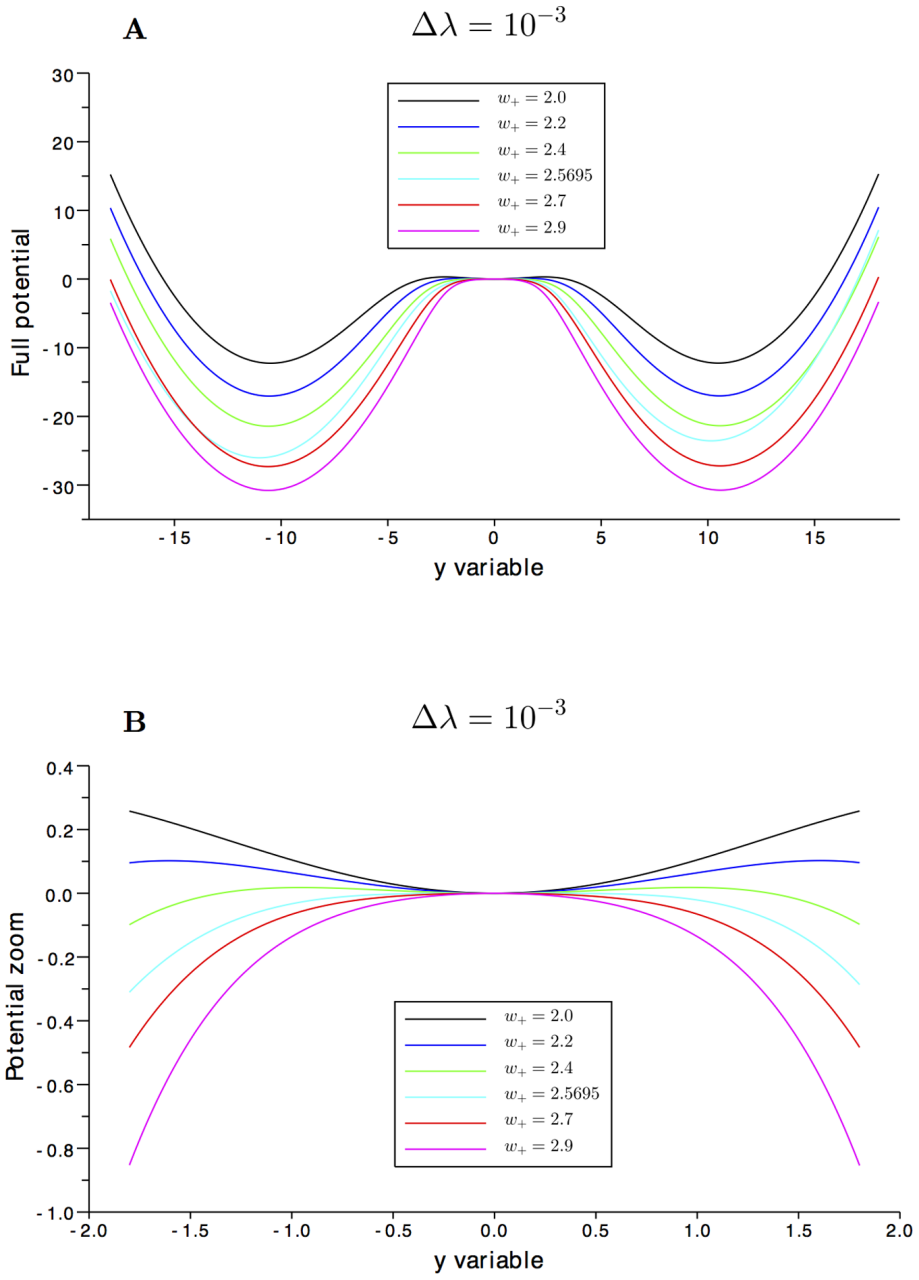
The 1D potential. **A**: Variation of the approximated 1D potential traced along the 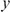 variable, varying 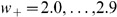 and for the biased case 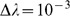. **B**: A zoom around the spontaneous state. Increasing 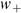, the central equilibrium point passes from a minima to a maxima.

Once we have found the slow manifold approximation 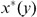, we can restrict the dynamics in (1) to a single effective Langevin equation. This equation is determined by a potential 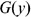 obtained from the evaluation of the dynamics over the slow manifold approximation, leading to
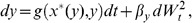
with 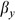 properly obtained in terms of 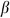 via the change of variables. The effective potential is determined by the relation 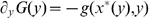. The 1D effective computed potential 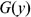 for the biased case (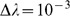) with respect to the slow variable 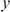 is plotted in Figure 4 for 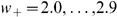 (left), together with a zoom at the spontaneous state (right). We note that increasing 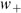 beyond the second bifurcation point, the spontaneous state pass from local minimum to local maximum. We remind that the effective potential 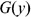 is given by formula (8) based on the approximated slow manifolds computed in Figure 3.

**Figure 5:**
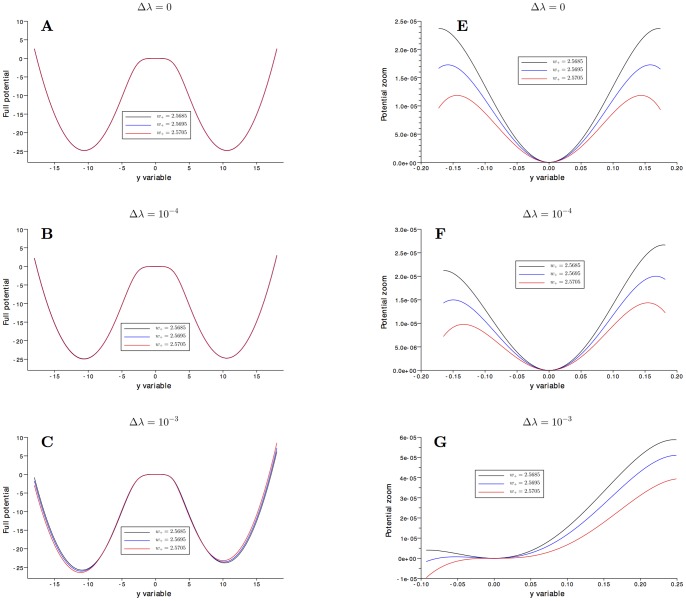
Potential at the bifurcation parameter 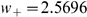. **A**: 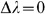, **E**: zoom for 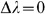 around the spontaneous state, **B**: 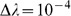, **F**: zoom for 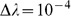 around the spontaneous state, **C**: 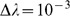, **G**: zoom for 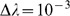 around the spontaneous state. For large bias 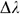 the potential becomes asymmetric, and for 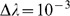 the spontaneous state changes its nature from a minima to a maxima.

The 1D effective computed potentials 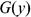 both for the unbiased 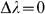 and the biased case 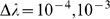 with respect to the slow variable 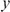 are plotted in Figure 5 for the three values of 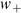 close to the second bifurcation point (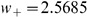, 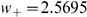, 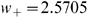). We note the asymmetry of the effective potential 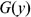 when increasing the bias 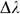 and the change in the nature of the spontaneous state when increasing the excitatory coefficient 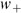.

This 1D effective potential can be considered as a good approximation of the equilibrium state of the 2D Fokker-Planck equation (associated to the system (1)) projected on the 1D-slow manifold which is parameterized by the slow variable 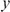, see Figure 3. We note that 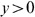 is pointing toward 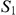 while 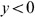 is pointing toward 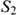, see Figure 2. Therefore, the top left (respectively low right) well whose minimum is attained at the stable decision state 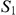 (respectively 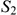) in the 2D 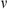-plane in Figure 2 corresponds to the right (respectively left) well in Figure 5.

**Figure 6:**
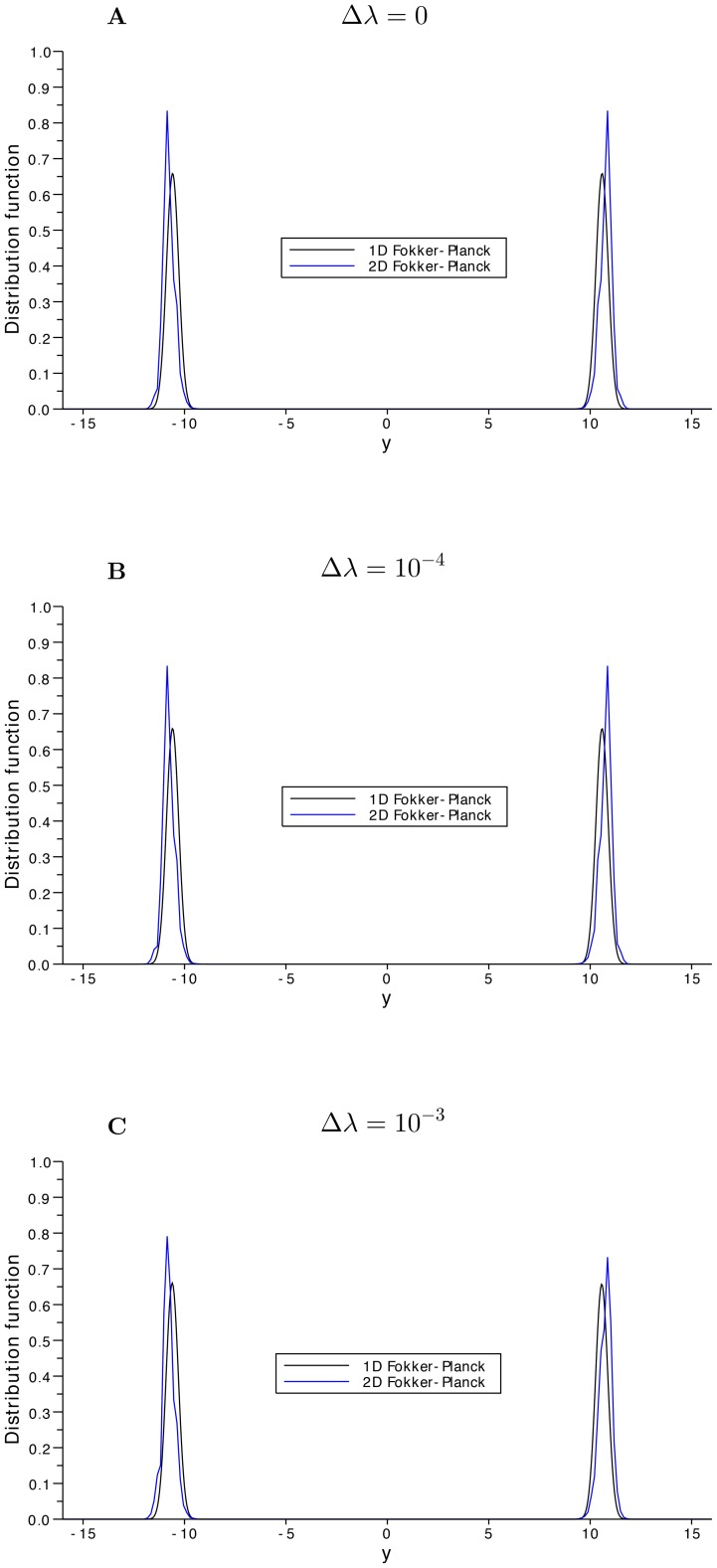
Distribution function comparison along 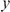. The computed marginals of the 2D problem (blue line) at time 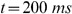 and the corresponding solution of the 1D reduced problem (black line) at the same time show a good agreement. In this test we have chosen the bifurcation parameter 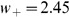, the standard deviation 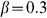 and the bias 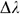 takes the values 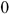 in **A**, 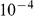 in **B** and 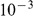 in **C**.

In Figure 6, we have computed the marginals along 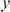 of the 2D numerical computations of the Fokker-Planck dynamics for 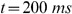, compared with the solution of the 1D reduction Fokker-Planck evolution equation (7) at 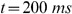. We observe the errors committed by the 1D reductions and we also observe that the slow-fast behavior is also present in the 1D reduced Fokker-Planck equation. However, since the one dimensional reduction is easily solved by numerical methods, then we can achieve much larger computational times and compute explicitly the approximated stationary state.

Concerning the comparison between the numerical solutions of the 2D complete Fokker-Planck and those of the 1D reduction, we refer the reader to [14]. We just note here that since the standard deviation is rather small (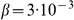), the stable states tends to be Dirac masses and the computational costs are unbearable to compute 2D solutions approaching the steady state. Note also that the 1D Fokker-Planck reduction can be solved numerically applying an implicit in time scheme, drastically reducing computational time costs.

### Bifurcation: Reaction Time and Performance

Finally, once the 1D reduction is validated by the above arguments, the biologically interesting quantities to compute are the performance 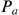 and the reaction times 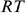. The first one corresponds to the ratio of good answers at equilibrium, and the second one to the smallest time needed to give an answer, no matter wether it is good or wrong. The computations of the reaction time 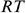 and performances 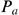 use the formulas in the supplementary material in [10], which hold locally around the spontaneous point and are based on the knowledge of the effective potential 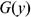.

**Figure 7:**
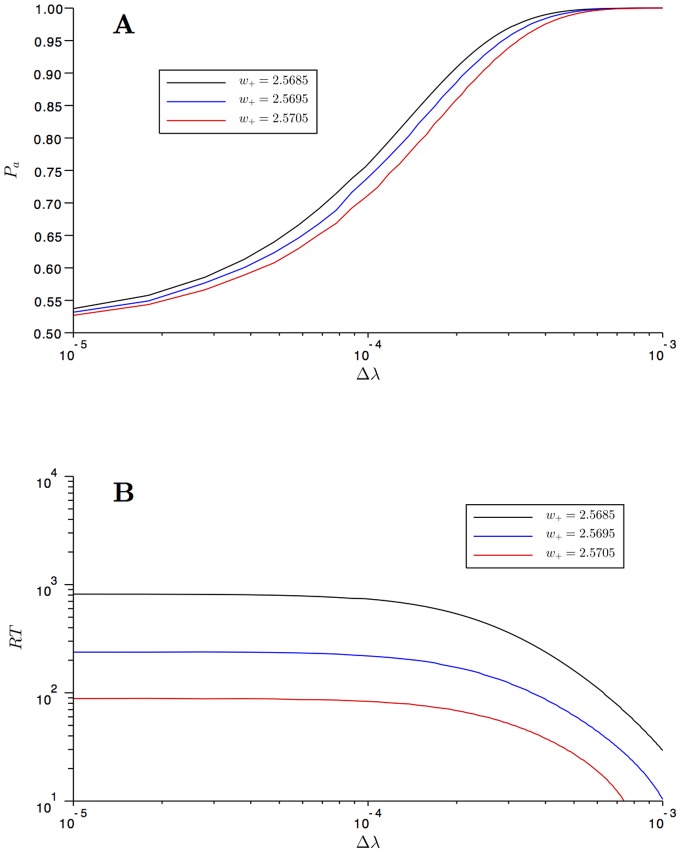
Performances and Reaction Times. **A**: the performances (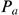) computed for three values of the bifurcation parameter 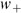 close to the one of the spontaneous state and as a function of the bias 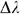. When 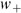 increases, the performance decreases, and it converges to 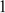 for large values of the bias 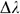. **B**: the reaction times (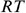) computed for three values of the bifurcation parameter 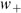 close to the one of the spontaneous state and as a function of the bias 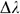. When 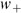 increases, the reaction time decreases. In both pictures the standard deviation is 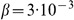. These behaviors are in agreement with previous results and experimental data.

In Figure 7 we plot the performance 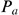 and reaction time 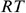 for 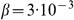 as a functions of 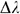 and 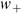. They are computed for the decision state with higher probability, the one for negative values of the 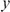-variable in Figure 5, and over an interval 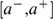 where 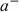 and 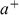 correspond to the 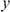-coordinates to the left and right of the spontaneous state for which 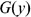 has a maximum. We observe the same qualitative behavior as in the comparisons with experimental data performed in [10]. As the bias increases, the potential gets tilted towards the preferred decision state, and thus the performance of the solution gets higher. Accordingly, the reaction time decreases as the bias increases.

## Discussion

Here, we have reinforced the link between the underlying physiology and the observed behavioral response in decision-making tasks by developing a new strategy for the 1D reduction of the dynamics of the neuronal circuit. In this way we have derived from the underlying detailed neuronal dynamics a “nonlinear” diffusion model valid for a wide range of the bifurcation parameter. We observe how our method recovers the reduced one dimensional dynamics proposed by Roxin and Ledberg in [10] close to the bifurcation point since we keep the same qualitative behavior in terms of performance and reaction times. We have also shown that our reconstructed effective potential leads to a good approximation of the stationary state of the two dimensional dynamics projected on the slow manifold. This reduction allows for efficient computations of the dynamics as soon as the approximated slow manifold is well-defined.

## Materials and Methods

In this section, we give more details of the 1D reduction of system (1) presented above. Preliminary results of this strategy were previously reported in [14] in the easier case of the supercritical Hopf bifurcation. The slow-fast behavior of the system (1) with no noise, i.e. 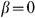, can be characterized by the fact that the jacobian of the linearized system at the unstable critical point 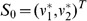 has a large condition number. More precisely, we write the deterministic part of the dynamical system (1) as 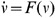, where 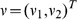 is the rate vector and 
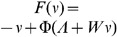
 is the drift. The linearized jacobian matrix at any equilibrium point is given by:
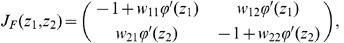
(2)where we have denoted by 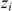 the values 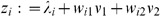. Since 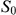 is a hyperbolic fixed point (saddle point), the jacobian 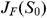 has two real eigenvalues, 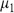 and 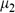 of opposite signs. Let us denote by 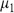 the (large in magnitude) negative eigenvalue and by 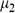 the (small) positive eigenvalue of 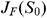. The small parameter 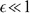 responsible for the slow-fast behavior is determined by the ratio



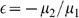



In order to reduce the system we need to introduce new variables based on the linearization of the problem. We will denote by 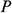 the matrix containing the normalized eigenvectors of 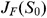 and by 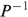 its inverse matrix. Furthermore, using the Hartman-Gro*β*man theorem [15], we know that the solutions of the dynamical system are topologically conjugate with its linearization in the neighborhood of a hyperbolic fixed point. Let us write it as follows
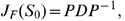
(3)where 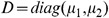 is the associated diagonal matrix. We can describe the coordinates 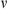 in the eigenvector basis and centered on the saddle point 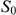 as follows:



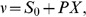



which gives the definition for the new variable 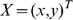, see also Figure 2, 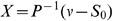


In these new coordinates 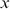 corresponds to the fast scale while 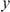 is the slow varying variable. We can conclude that system (1) reads in the 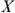 phase space as 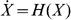 where 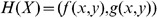 is the two dimensional vector defined by 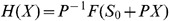, and thus,
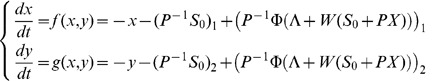
(4)


Direct computations gives that 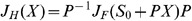, and using (3) and that 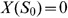, we obtain that 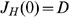. We can choose a new time scale for the fast variable 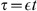 in such a way that for 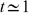, then 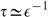. Then, the fast character of the variable 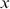 is clarified and the system reads as
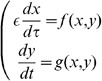



Our claim is that the curve of equation 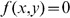 approximates well when 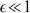 the slow manifold, that is the unstable manifold that joins the spontaneous point 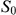 to the other equilibrium points. We refer the reader to the discussion in [12] on stochastic slow-fast dynamics. Due to the non-linearity of the function 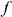 in (4), we cannot expect an explicit formula for the solution of this equation. Nevertheless, since 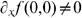, the resolution in the neighborhood of the unstable equilibrium point 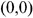 is insured by the implicit function theorem. Hence we can define a curve:
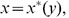
(5)


such that 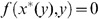 in a neighborhood of 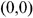. We note also that, by construction the approximated slow manifold 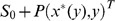, implicitly defined by (5), intersects the exact slow manifold at all equilibrium points, i.e. where both 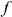 and 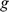 vanishes. Finally, we can conclude the slow-fast ansatz, replacing the complete dynamics by the reduced dynamics on the approximated slow manifold, and obtain the reduced one dimensional differential equation:
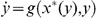



A similar treatment can be done in the presence of the noise terms. By changing variables from 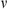 to 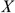 and since the new variables 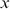 and 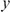 are linear combination of 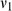 and 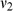, then it is standard to check
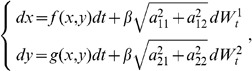
where 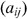 are the elements of the matrix 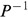 and 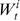 are two independent normalized white noise. Arguing as in the deterministic case, we can choose a fast time scale for the variable 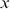 and we note that the noise term also changes since 
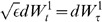
 and 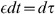. Therefore, we can deduce again that the reduced one dimensional model must read:



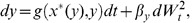
(6)with 
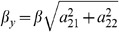
.

Finally, we can consider the Fokker-Planck or forward Kolmogorov equation associated to the 1D stochastic differential equation (6). This gives the reduced dynamics for the probability density 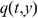 of finding neurons with rate determined by the rate 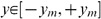 over the approximated slow manifold 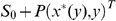, for 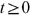. Actually, it must obey to the following 1D Fokker-Planck equation:
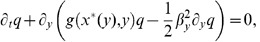
(7)


with no-flux boundary conditions on 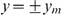: 
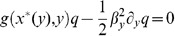
, see [16] and [14]. The 1D Fokker-Planck dynamics (7) are given for large times by the stationary solution given by the invariant measure for the stochastic differential equation (6). We can easily find it by defining the associated potential 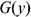 being the antiderivative of the flux term 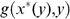. In other words, we can always define the potential function:
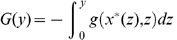
(8)


Then, the stationary probability density of the 1D Fokker-Planck dynamics (7) is given by
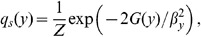
where 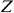 is the normalization constant. As explained also in [17], this stationary solutions are the asymptotic equilibrium states for the solution of the Fokker-Planck equation. In other words, letting time going to infinity, the solution 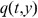 to (7) must converge to 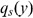. We have shown in [17] that the decay to equilibrium for the two dimensional problem was exponential. This rate of convergence is also true for the 1D reduction since the potential is a small perturbation of convex potentials, see the well-know results in [18], [19]. We also recall that we are interested in the long time behavior of the solutions and that the convergence in the slow manifold given by the variable 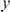 is slow. Hence it is relevant to have a direct computation of their asymptotic behavior without need to solve the whole 1D or 2D Fokker-Planck equation.

We also remind that the solution of the system (1) is related to the 2D Fokker-Planck or forward Kolmogorov equation:

with boundary conditions 
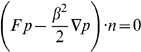
 on the boundary of the domain 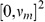, see [16]. Let us remark that, once we know the stationary state of the 1D reduced Fokker-Planck equation (7), we can approximate the long-time dynamics of all quantities of interest related to the system (1) and the 2D Fokker-Planck (9). More precisely, as 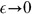, 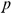 approaches a concentrated density along the the curve 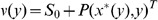. Then, for any test function 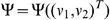, the moment 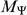 of the stationary probability distribution function 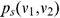 as 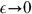 can be approximated by
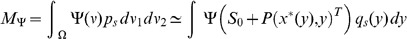



This formulae can be used to compute either classical moments of 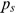 or marginals.
